# A comparison of the clinical, laboratory and epidemiological features of two divergent subpopulations of *Plasmodium knowlesi*

**DOI:** 10.1038/s41598-021-99644-8

**Published:** 2021-10-11

**Authors:** Ting Huey Hu, Nawal Rosli, Dayang S. A. Mohamad, Khamisah A. Kadir, Zhen Hao Ching, Yaw Hung Chai, Nur Naqibah Ideris, Linda S. C. Ting, Adeline A. Dihom, Sing Ling Kong, Edmund K. Y. Wong, Jenny E. H. Sia, Tiana Ti, Irene P. F. Chai, Wei Yieng Tang, King Ching Hii, Paul C. S. Divis, Timothy M. E. Davis, Cyrus Daneshvar, Balbir Singh

**Affiliations:** 1 Malaria Research Centre, Universiti Malaysia Sarawak, Kota Samarahan, Malaysia; 2 Kapit Hospital, Kapit, Sarawak Malaysia; 3 University of Western Australia, Medical School, Fremantle, WA Australia; 4 Department of Respiratory Medicine, University Hospitals Plymouth NHS Trust, Plymouth, UK

**Keywords:** Malaria, Epidemiology

## Abstract

*Plasmodium knowlesi*, a simian malaria parasite responsible for all recent indigenous cases of malaria in Malaysia, infects humans throughout Southeast Asia. There are two genetically distinct subpopulations of *Plasmodium knowlesi* in Malaysian Borneo, one associated with long-tailed macaques (termed cluster 1) and the other with pig-tailed macaques (cluster 2). A prospective study was conducted to determine whether there were any between-subpopulation differences in clinical and laboratory features, as well as in epidemiological characteristics. Over 2 years, 420 adults admitted to Kapit Hospital, Malaysian Borneo with knowlesi malaria were studied. Infections with each subpopulation resulted in mostly uncomplicated malaria. Severe disease was observed in 35/298 (11.7%) of single cluster 1 and 8/115 (7.0%) of single cluster 2 infections (*p* = 0.208). There was no clinically significant difference in outcome between the two subpopulations. Cluster 1 infections were more likely to be associated with peri-domestic activities while cluster 2 were associated with interior forest activities consistent with the preferred habitats of the respective macaque hosts. Infections with both *P. knowlesi* subpopulations cause a wide spectrum of disease including potentially life-threatening complications, with no implications for differential patient management.

## Introduction

The simian malaria parasite, *Plasmodium knowlesi* was found to be the commonest cause of human malaria infections in the Kapit Division of Sarawak, Malaysian Borneo in 2004^1^. Subsequent studies have shown that zoonotic malaria cases occur throughout Southeast Asia and in the Andaman and Nicobar islands of India^2–5^. The highest incidence is in Malaysia, where 13,612 knowlesi malaria cases were reported between 2017 to 2020, with 87% from the Malaysian Borneo states of Sabah and Sarawak (B. Singh, unpublished data). All the indigenous malaria cases in Malaysia in 2018 and 2019 were due to *P. knowlesi*^6^. Human knowlesi malaria infections have been increasing in Malaysia since they were first reported in 2004^1^. From the 120 cases detected in Sarawak over a 32-month period at that time^1^, the annual number of reported cases in Malaysia has increased to 912 in 2009, and to between 1600 and 4131 since 2012^7^. This increase may reflect improved diagnostic capacity, decrease in cross-species immunity due to decrease in malaria caused by human malaria species, and increased interaction between humans, macaques and mosquito vectors. This latter explanation is based on land-use changes leading to alterations in mosquito abundance and composition, and to greater proximity of the movement of the reservoir macaque hosts to human habitation^7,8^. The increase in zoonotic malaria cases is of public health concern and a threat to the elimination of malaria.

Micro-satellite genotyping of *P. knowlesi* isolates from wild macaques in Kapit, Malaysian Borneo and humans across Malaysia has identified two simian host-associated genetically distinct subpopulations^9^. Two-thirds of human infections were of one subpopulation (termed cluster 1) associated with long-tailed macaques (*Macaca fascicularis*), while the second subpopulation (cluster 2) was associated with pig-tailed macaques (*Macaca nemestrina*). Subsequent whole genome sequence analysis of *P. knowlesi* isolates confirmed these two subpopulations in Malaysian Borneo and further identified a third distinct subpopulation (cluster 3) occurring in Peninsular Malaysia^10,11^. Microsatellite genotyping of additional human and macaque samples from Sarawak, Sabah and Peninsular Malaysia confirmed these divergent *P. knowlesi* subpopulations across the three regions^12^. A recent study, using allele-specific PCR to define further the molecular epidemiology of subpopulations, identified cluster 1 infections to be the commonest in all locations across Malaysian Borneo^13^. Furthermore, it showed that, amongst 1204 knowlesi malaria patients admitted to Kapit Hospital between 2000 and 2018, cluster 1 accounted for two-thirds of cases with no evidence of seasonal or temporal influence on this proportion.

The Kapit Division of Sarawak is mainly composed of primary and secondary forests, with over 600 rural communities living in longhouses accommodating between 5 and over 50 families. These longhouses are situated on river banks in close proximity to forests which are the habitats of long-tailed and pig-tailed macaques. Long-tailed macaques are found in primary, secondary and riparian forests, while pig-tailed macaques mainly prefer to live and forage in primary forests^14,15^. In camera trapping surveys conducted in 5 localities in Sarawak, Mohd-Azlan et al*.* noted that pig-tailed macaques had a higher occupancy in both primary and secondary forests compared to long-tailed macaques^16^. Furthermore, long-tailed macaques were more likely to be found in open habitats such as Beach and Kerangas forests than pig-tailed macaques. There are currently no data relating to the relative abundance of the long-tailed and pig-tailed macaques in Sarawak. The higher proportion of patients seen at Kapit Hospital with cluster 1 *P. knowlesi* infections may be due to differences in abundance of the reservoir macaque hosts or to rural communities predominantly conducting activities such as subsistence farming and fishing in close proximity to their longhouses, and overlapping with the preferred habitats of long-tailed macaques. *P. knowlesi* parasites appear in the blood between 7 and 9 days after a person has been bitten by an infected mosquito^17^, so a history of travel within 14 days of hospital admission would indicate the most likely place a patient acquired the infection.

In our first prospective clinical study of 107 cases of knowlesi malaria presenting to Kapit Hospital from 2006 to 2008, we found that *P. knowlesi* infections covered a wide spectrum of disease. Although most cases presented with uncomplicated infections, 6.5% were severe and there was a case fatality rate of 1.8%^18^. Subsequent studies in Sarawak^19^ and Sabah^20,21^ have also found mainly uncomplicated disease, and have identified older age, higher parasitaemia and proportions of schizonts, thrombocytopenia, abdominal pain and breathlessness as features associated with severe infections. Not all *P. knowlesi* infections result in disease as shown by community-based studies which have identified asymptomatic individuals with *P. knowlesi* infections in Vietnam^22^, Malaysian Borneo^23,24^ and Peninsular Malaysia^25^.

The recognition of two divergent subpopulations of *P. knowlesi* from specific macaque hosts raises the possibility that they represent two independent zoonoses with distinct clinical and laboratory features^26^. Therefore, a prospective study was undertaken at Kapit Hospital to determine whether i) the baseline characteristics and clinical course of patients differ by *P. knowlesi* subpopulation, and ii) any between-subpopulation differences have implications for clinical management. In addition, demographic and behavioural data were obtained from each patient to determine whether there were any between-subpopulation epidemiological differences.

## Results

### Baseline characteristics

**Figure 1:**
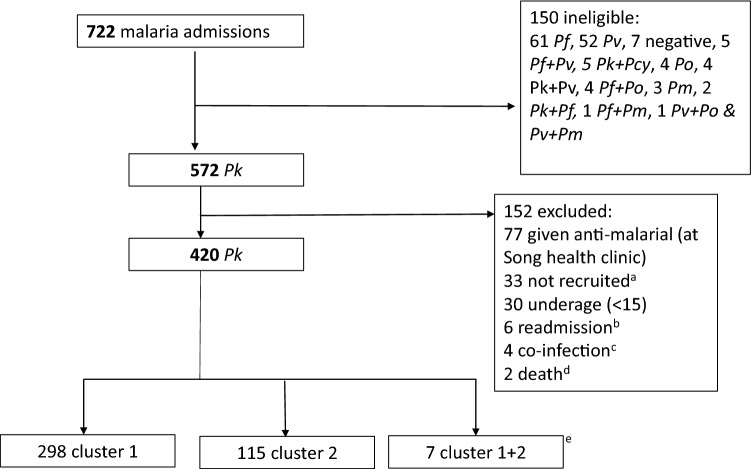
Recruitment flow chart of patients at Kapit Hospital from 27th Sept 2016 until 12th Oct 2018. ^a^Patients not recruited since study clinicians not working and/or not informed (33), ^b^Only index cases were recruited, ^c^Other concurrent infections such as active tuberculosis (n = 1), leptospirosis (n = 2) and dengue positive by rapid diagnostic test (n = 1) and ^d^Not recruited; PCR analysis not done for one patient who was identified posthumously as *P. knowlesi* by microscopy while another was identified as *P. knowlesi* after transfer to a tertiary hospital (patient's sample obtained and subsequently identified by PCR). ^e^Patients with both cluster 1 and cluster 2 infections. *Pk* = *P. knowlesi*; *Pm* = *P. malariae*; *Pf* = *P. falciparum*, *Pv* = *P. vivax*, Po = *P. ovale*.

Of 722 admissions with a diagnosis of malaria, there were 420 eligible patients with PCR confirmed *P. knowlesi* monoinfection (Fig. 1). These patients had a median [IQR] age of 42 [31–57] years, most were male (61.4%) and Iban was the dominant ethnicity (86.2%) (Table 1). Single *P. knowlesi* subpopulations were identified in 413/420 patients (98.3%), of which 298/413 (72.2%) cases were cluster 1 and 115/413 (27.8%) cluster 2. While there was no significant age difference between subpopulations, patients with cluster 2 infections were more likely to be male (*p* = 0.043) and to have reported recent jungle activity (*p* = 0.023) than those with cluster 1. After adjusting for age, types of activities remained associated with subpopulations, with patients with cluster 1 infections 1.8-fold (95% CI 1.14–2.95, *p* = 0.013) more likely to report peri-domestic activity than those with cluster 2 (Table 2). The types of activities were sex- and age-specific, with females being fivefold (OR 5.09 (95% CI 3.09–8.62), *p* < 0.001) more likely to report peri-domestic activity compared to males and each year increase in age increasing the odds of reported peri-domestic activity (OR 1.04 (95% CI 1.03–1.06), *p* < 0.01) (see Supplementary Table S1 online).

**Table 1:** Demographics and clinical presentation of *P. knowlesi*.

	All patients	Patients with cluster 1 infection	Patients with cluster 2 infection	*p* value
No. patients	420	298 (72.2)	115 (27.8)	–
Age, (years)	42 [31–57]	44 [29–59]	41 [31–56]	0.546
Male	258 (61.4)	172 (57.7)	79 (68.7)	**0.043**
Iban ethnicity	362 (86.2)	265 (88.9)	91 (79.1)	**0.016**
**Types of activities**				
Jungle	172 (41.0)	110 (36.9)	58 (50.4)	
Peri-domestic	240 (57.1)	183 (61.4)	54 (47.0)	
Peri-domestic & jungle	8 (1.9)	5 (1.7)	3 (2.6)	**0.023**
Previous malaria	42 (10.0)	28 (9.4)	12 (10.4)	0.715
**Referral route**				
Directly presented to hospital	304 (72.4)	224 (75.2)	77 (67)	
Given anti-malarial at referral health center	116 (27.6)	74 (24.8)	38 (33)	0.108
I.V artesunate treatment at referral health center	3 (0.7)	1 (0.3)	2 (1.7)	0.087
Severe disease	44 (10.5)	35 (11.7)	8 (7)	0.208
Duration of illness (Days)	3 [2–5]	3 [2–5]	4 [2–6]	0.111
Pulse Rate (beats/min)	100 [88–112]	101 (± 18)	99 (± 19)	0.404
Respiratory Rate (breaths/min)	21 [20–23]	21 [20–23]	21 [20–23]	0.270
Mean arterial pressure (mm/Hg)	93.8 (± 13.5)	93.2 (± 13.9)	95.2 (± 12.5)	0.166
Oxygen saturations (%)	98 [97–99]	98 [97–99]	98 [97–99]	0.165

Data are presented as no. (%), mean (± SD) or median [IQR]. *p* value < 0.05 are in bold. Adjusted Fisher’s exact from pairwise Bonferroni correction remained significant (*p* = 0.039) between jungle and peri-domestic activities.

**Table 2:** Association of *P. knowlesi* clusters with activities and age in *P. knowlesi* patients (N = 405).

Dependent: clusters	Cluster 2 (baseline)	Cluster 1	OR (95% CI) (univariable)	*p* value	OR (95% CI) (multivariable)	*p* value
**Activity, no. (%)**						
Jungle	58 (51.8)	110 (37.5)	1		1	
Peri-domestic	54 (48.2)	183 (62.5)	1.79 (1.15–2.78)	**0.010**	1.83 (1.14–2.95)	**0.013**
**Age, years**						
Mean (SD)	42.7 (15.2)	44.2 (18.5)	1.00 (0.99–1.02)	0.443	1.00 (0.98–1.01)	0.820

*p* < 0.05 in bold.

The median duration of illness before presentation and route of hospital referral in the two groups were similar (Table 1). Of the 27.6% patients who received treatment before recruitment, intravenous artesunate was given to one patient with a cluster 1 infection (0.3%) and two patients with cluster 2 infections (1.7%). Most cases were uncomplicated with 44/420 (10.5%) defined as severe according to WHO criteria^27^. There were seven patients with mixed cluster 1 and 2 infections and all were men. Their median [IQR] age was 42 [36–48] years and one had severe disease characterised by hyperparasitaemia, jaundice, acute kidney injury (AKI) and severe anaemia.

### Laboratory findings

Haematological and biochemical findings at presentation were largely similar in the subpopulation groups, with thrombocytopenia being the commonest and almost universal abnormality (Table 3). Patients with cluster 1 infections had lower median platelet counts (*p* < 0.001) and serum sodium concentrations (*p* = 0.031), and had higher serum total bilirubin (*p* = 0.049) and blood urea concentrations (*p* = 0.002), than those with cluster 2 infections (Table 3). These differences persisted after adjustment for age, sex, and ln (parasitaemia) (*p* < 0.05; Supplementary Table S2 online), but were all of relatively minor clinical significance.

**Table 3:** Baseline laboratory findings of patients with *P. knowlesi* infections.

	Reference ranges	All patients (N = 420)	Patients with cluster 1 infections (N = 298)	Patients with cluster 2 infections (N = 115)	*p* value
Haemoglobin (g/dL)	12–17	13.3 [12.0–14.6]	13.2 [12–14.7]	13.5 [13–14.2]	0.201
White blood cell count (× 10^3^/µL)	4–10	6.1 [5.0–7.2]	6.1 [5–7.3]	6.2 [5.2–7.1]	0.978
Platelet count (× 10^3^/µL)	150–400	69 [46–104]	64 [41–97]	81 [55–113]	**< 0.001**
Thrombocytopenic (%)	< 150 × 10^3^ platelets/µL	375 (93.5)	275 (95.2)	95 (89.6)	0.06
Prothrombin time (s)	9.8–14.8	13.5 [12.2–15.4]	13.7 [12.3–15.7]	13.3 [11.9–14.7]	**0.042**
Serum creatinine (µmol/L)	63–133	92 [80–107]	92 [80–109]	92 [80–106]	0.897
Serum urea (mmol/L)	1.7–8.3	4.9 [3.9–6.4]	5.0 [4.0–7.1]	4.6 [3.5–5.4]	**0.002**
Serum total bilirubin (µmol/L)	< 17	20.6 [15.–30.0]	21.4 [15.4–31.2]	19.2 [14.2–25.1]	**0.049**
Serum alanine aminotransferase (IU/L)	< 41	38 [24–61]	38 [24–60]	37 [23–66]	0.790
Serum albumin (g/dL)	35–60	39 [37–41]	39 [36–41]	40 [38-41]	0.071
Serum sodium (mmol/L)	136–152	133 [130–135]	133 [130–135]	133 [131–136]	**0.031**
Serum glucose (mmol/L)	3.1–6.4 (fasting)	6.4 [5.7–7.5]	6.4 [5.75–7.6]	6.2 [5.5–7.3]	0.191
Plasma lactate (mmol/L)	< 2.5	1.1 [0.8–1.5]	1.1 [0.8–1.5]	1.1 [0.8–1.7]	0.345
Parasite count (parasites/µL)		1439 [279–6223]	1250 [255–6100]	1797 [346–5727]	0.832
Parasite count > 20,000 (parasites/µL)		49 (11.7)	32 (10.7)	15 (13)	0.494
Parasite count > 100,000 (parasites/µL)		17 (4)	12 (4)	4 (3.5)	1.000
Geometric mean of parasite count (parasites/µL)		1450 (± 9)	1420 (± 7)	1455 (± 9)	0.922

Data are presented as no. (%), mean (± SD) or median [IQR]. *p* value < 0.05 are in bold. *p* value is between cluster 1 and cluster 2.

The median parasite count for all *P. knowlesi* cases was 1439 [279–6223]/µL (Table 3) and was similarly distributed by subpopulation. Cluster 1 had a median parasite count of 1250 [255–6100]/µL, with 32/298 (10.7%) > 20,000/µL and 12/298 (4%) > 100,000/µL. Cluster 2 had a median parasite count of 1797 [346–5727]/µL, with 15/115 (13%) > 20,000/µL and 4/115 (3.5%) > 100,000/µL. There was no significant difference between the two subpopulations in the geometric mean parasitaemia (*p* = 0.922). In the seven patients infected with both cluster 1 and 2 subpopulations, the median parasite count was similar to those in single subpopulation infections, specifically 1488 [752–19,319] parasites/µL with 2/7 (28.6%) > 20,000/µL and 1/7 (14.3%) > 100,000/µL.

### Predictors of type of *P. knowlesi* subpopulation

Optimal thresholds for platelet count, serum concentrations of sodium, total bilirubin and urea controlled for age, sex and ln (parasitaemia) in multivariable logistic regression remained independently significant for differences between subpopulations (see Supplementary Table S3 online). However, the AUCs for all variables were < 0.7.

### Severe malaria

**Figure 2:**
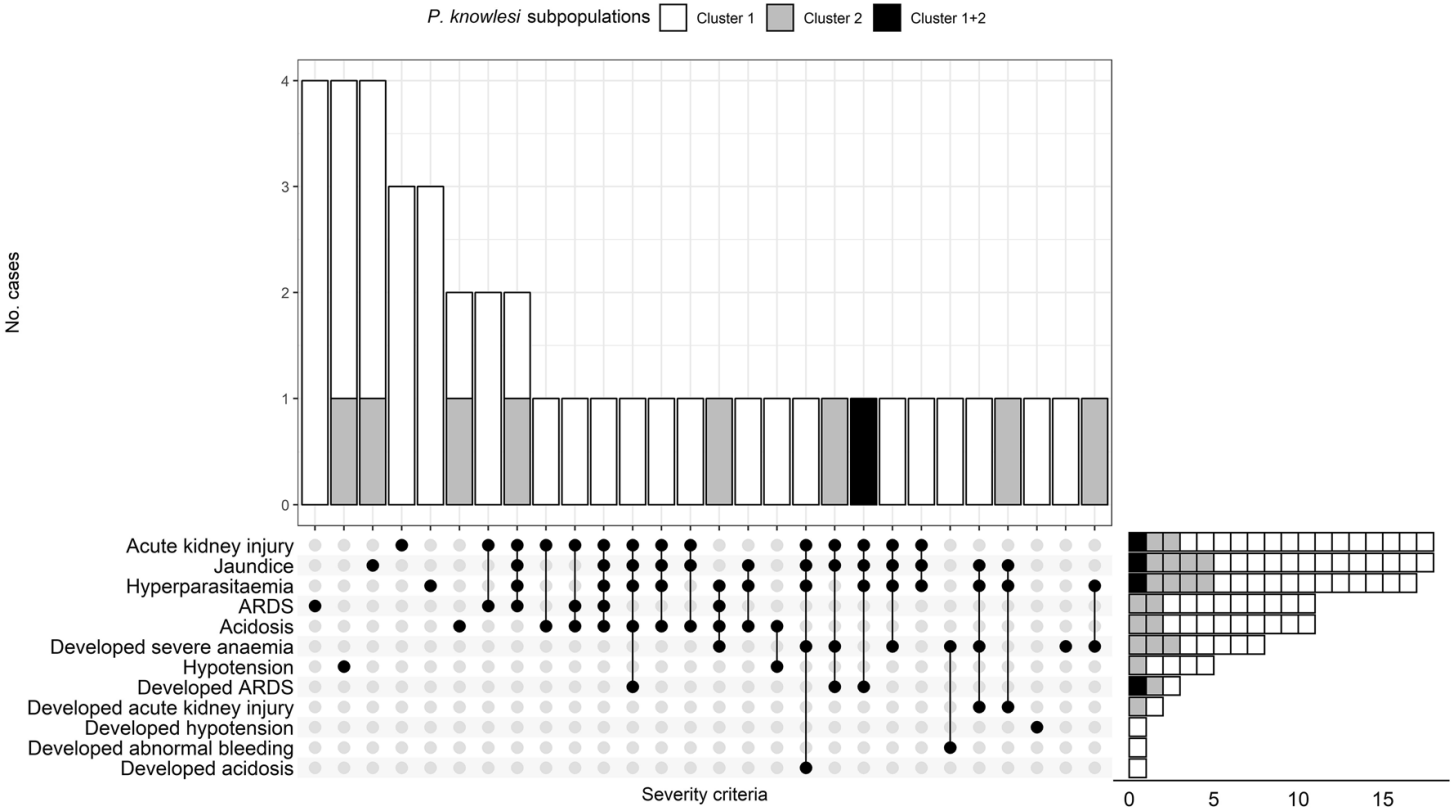
Combinations of severe criteria among patients infected with *P. knowlesi* subpopulations (N = 44). The scale on the far right x axis shows the total number of patients with each severe criteria. The y-axis of the graph shows number of patients with same combination/s of severe criteria. The criteria were : hyperparasitaemia (> 100,000 parasites/μL); severe anaemia (haemoglobin concentration < 7 g/dL); hypotension (systolic blood pressure < 80 mmHg); acute kidney injury (serum creatinine > 265 μmol/L or blood urea > 20 mmol/L); jaundice (serum total bilirubin > 50 μmol/L) with parasite count > 20,000/μL; acidosis (base deficit > 8 mEq/L or plasma bicarbonate < 15 mmol/L or venous plasma lactate ≥ 5 mmol/L); acute respiratory distress syndrome (ARDS) (respiratory rate > 30 breaths/minute plus oxygen saturation < 92% on air and/or pulmonary infiltrates on chest radiograph).

On the basis of WHO research criteria^27^, we identified 44 cases of severe malaria, with AKI being the commonest complication. Of these, 22 (50%) met 2 or more severe criteria (Fig. 2). The commonest criterion to develop after admission was severe anaemia, occurring in eight patients including three cases that were uncomplicated at presentation (Table 4). Out of these eight patients, 5 had hyperparasitaemia, 2 had a parasite count > 20,000/µL and one patient had a parasite count of 243/µL but with abnormal bleeding^28^. No patients presented with, or developed, hypoglycaemia or met the criteria for cerebral malaria. Blood cultures were taken from six severely ill patients with clinical features of sepsis. All these patients were culture negative but received empirical intravenous antibiotic therapy (ceftriazone in three cases, metronidazole plus cefoperazone in two with clinical features of intra-abdominal infection, and amoxycillin/clavulanate in one). A higher incidence of severe disease was present in patients with cluster 1 infections (11.7% [95% CI: 8.3–16%] versus 7.0% [95% CI 3.1–13.3%] in cluster 2) but this did not reach statistical significance (*p* = 0.208; Table 1). The severe cases with cluster 1 tended to have lower parasite counts compared to severe cluster 2 infections (geometric mean, 24,052 vs 89,322/µL, *p* = 0.098), with 50% (4/8) of severe cluster 2 cases having hyperparasitaemia (> 100,000/ µL) compared to 34% (12/35) in the severe cluster 1 group (*p* = 0.443) (Table 4).

**Table 4:** Demographics and characteristics among severe knowlesi malaria infections.

	All patients (N = 44)	Patients with cluster 1 infections (N = 35)	Patients with cluster 2 infections (N = 8)	*p* value
Male	16 (36.4)	12 (35.3)	3 (37.5)	1.000
Age (years)	54.3 (± 16.6)	55.3 (± 16.9)	51.9 (± 16.4)	0.410
Duration of illness (days)	4 [3–7]	3 [3–7]	6 [5–7]	0.170
Haemoglobin (g/dL)	11.6 [10.4–13]	11.9 [10.9–13]	10.5 [9.8–12]	0.121
Platelet count (× 10^3^/µL)	31 [19–51]	32 [16–50]	27 [20–51]	0.988
Parasite count (parasites/µL)	58,920 [19,649–139,643]	49,298 [7656–111916]	115,481 [36,568–220,831]	0.126
Parasite count range (parasites/µL)	243–293,290	243–293,290	25,997–252,532	–
Geometric mean of parasite count (parasites/µL)	31,888 (± 7)	24,052 (± 8)	89,322(± 3)	0.098
Parasite count > 100,000 (parasites/µL)	17 (38.6)	12 (34.3)	4 (50)	0.443
**WHO-defined severe criteria**				
ARDS	14 (31.8)	10 (28.6)	3 (37.5)	0.681
Hyperparasitaemia	17 (38.6)	12 (34.3)	4 (50)	0.443
Hypotension	6 (13.6)	5 (14.3)	1 (12.5)	1.000
Acute kidney injury	20 (45.5)	16 (45.7)	3 (37.5)	1.000
Jaundice	18 (40.9)	13 (37.1)	4 (50.0)	0.692
Acidosis	12 (27.3)	10 (28.6)	2 (25)	1.000
Severe anaemia	8 (18.2)	5 (14.3)	3 (37.5)	0.153
Abnormal bleeding	1 (2.3)	1 (2.9)	0	1.000
Hypoglycaemia	0	0	0	–
Cerebral malaria	0	0	0	–

Data are presented as no. (%), mean (± SD) and median [IQR] unless otherwise stated. One severe case had mixed infection with cluster 1 and cluster 2. *p* value is between cluster 1 and cluster 2. Hyperparasitaemia is defined as > 100,000 parasites/μL; severe anaemia—haemoglobin level < 7 g/dL; hypotension—systolic blood pressure < 80 mmHg; acute kidney injury—serum creatinine > 265 μmol/l or blood urea > 20 mmol/l; jaundice—serum bilirubin > 50 μmol/l with parasite count > 20,000/μl; hypoglycaemia—serum glucose < 2.2 mmol/l; acidosis—base deficit > 8 meq/l or plasma bicarbonate < 15 mmol/l or venous plasma lactate ≥ 5 mmol/l; ARDS (acute respiratory distress syndrome)—respiratory rate > 30 breaths/minute plus oxygen saturation < 92% on air and/or pulmonary infiltrates on chest radiograph; cerebral malaria (unarousable coma). Abnormal bleeding involved splenic bleeding.

There was no significant difference observed between the proportions of severity criteria met by cases of severe malaria in the two *P. knowlesi* subpopulations (Table 4). Among all knowlesi malaria cases, AKI was the commonest complication in cluster 1 patients, occurring in 5.6% (16/298) of cases, while for cluster 2 infections the commonest severe criteria were hyperparasitaemia and jaundice, both of which occurred in 3.5% (4/115) of cases (Table 5). Multivariable logistic regression using severe criteria as outcome among all knowlesi malaria cases showed no significant difference between the two clusters for any severe criteria (Supplementary Table S4 online). Subpopulation was not independently associated with severe disease (*p* = 0.427, see Supplementary Table S5 online).

**Table 5:** Overall distribution of severe criteria among patients infected with *P. knowlesi*.

Severe criteria	All patients (N = 420)	Patients with cluster 1 infections (N = 298)	Patients with cluster 2 infections (N = 115 )	*p* value
ARDS	14 (3.3)	10 (3.4)	3 (2.6)	1.000
Hyperparasitaemia	17 (4.0)	12 (4.0)	4 (3.5)	1.000
Hypotension	6 (1.4)	5 (1.7)	1 (0.9)	1.000
Acute kidney injury	20 (5.0)	16 (5.6)	3 (2.8)	0.300
Jaundice	18 (4.3)	13 (4.4)	4 (3.5)	0.789
Acidosis	12 (3.5)	10 (4.1)	2 (2.1)	0.521
Severe anaemia	8 (1.9)	5 (1.7)	3 (2.6)	0.691
Abnormal bleeding	1 (0.2)	1 (0.3)	0	1.000

Data are presented as no. (%). Hyperparasitaemia is defined as > 100,000 parasites/μL; severe anaemia defined as haemoglobin level < 7 g/dL; hypotension—systolic blood pressure < 80 mmHg; acute kidney injury—serum creatinine > 265 μmol/l or blood urea > 20 mmol/l; jaundice—serum bilirubin > 50 μmol/l with parasite count > 20,000/μl; hypoglycaemia—serum glucose < 2.2 mmol/l; acidosis—base deficit > 8 meq/l or plasma bicarbonate < 15 mmol/l or venous plasma lactate ≥ 5 mmol/l; ARDS (acute respiratory distress syndrome)—respiratory rate > 30 breaths/minute plus oxygen saturation < 92% on air and/or pulmonary infiltrates on chest radiograph. *p* value is between cluster 1 and cluster 2. Abnormal bleeding involved splenic bleeding.

**Figure 3:**
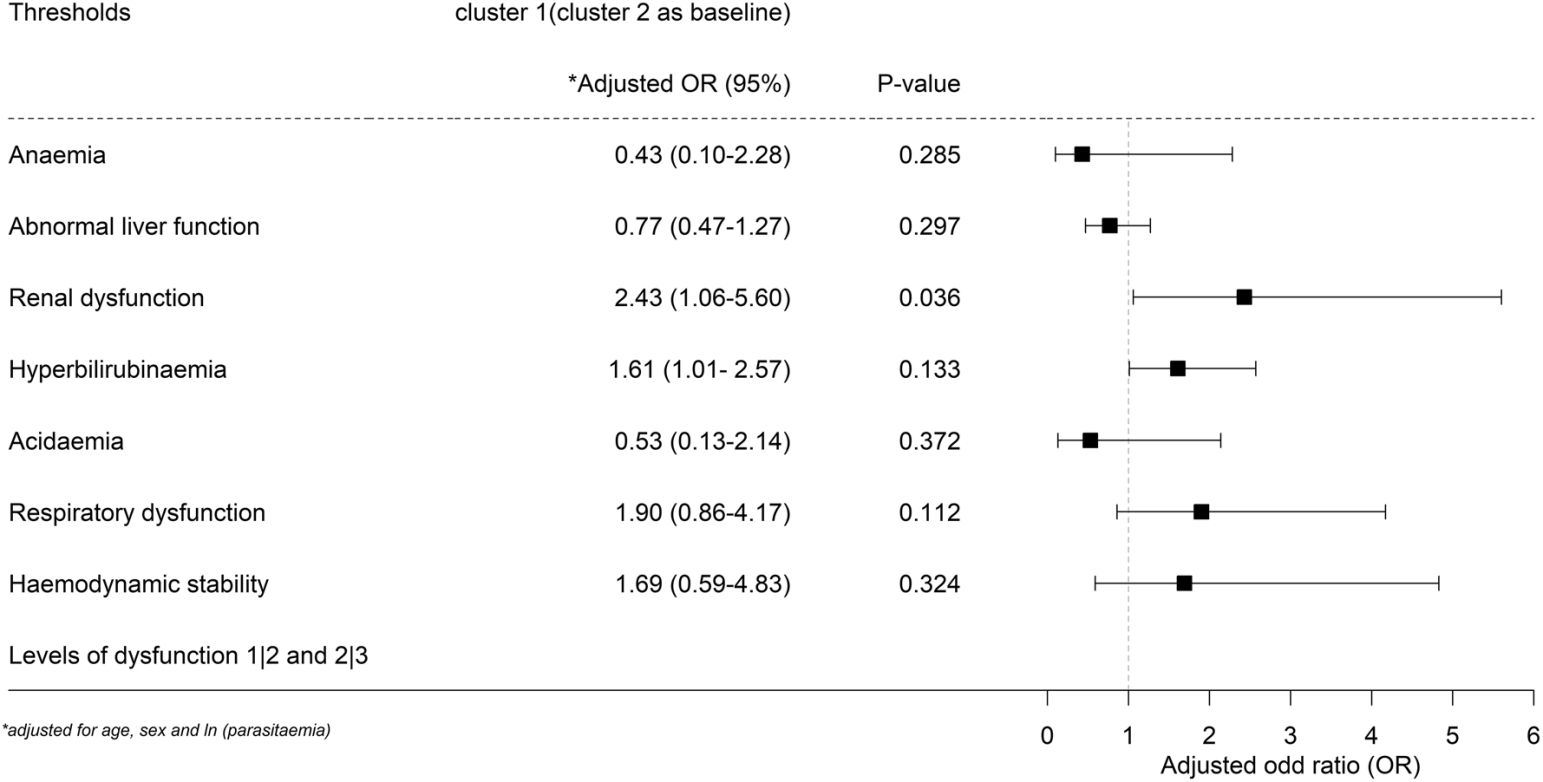
Forest plot with end organ markers as outcome in ordinal logistic regression (cluster 1 = 298, cluster 2 = 115) adjusted for age, sex and ln (parasitaemia) for *P. knowlesi* infection. Detailed categorisation of variables is summarised in Supplementary Table S7 online.

Ordinal regression was used to assess associations between cluster types and markers of end organ disease, and identified a significant association between clusters for renal dysfunction (Fig. 3). Cluster 1 infections showed 2.4-fold higher odds of step wise change from normal to abnormal and to severe renal dysfunction compared to cluster 2 infections (*p* = 0.036) after adjustment for age, sex and ln (parasitaemia).

### Clinical course

Overall 12 patients developed one or more severe criteria, with 4 developing anaemia (two from each cluster respectively with one cluster 1 uncomplicated upon presentation), 2 developed ARDS (cluster 1 and mixed cluster), 1 developed ARDS with anaemia (cluster 2), 1 developed anaemia and acidosis (cluster 1), 1 developed anaemia and abnormal bleeding (cluster 1, uncomplicated at presentation), 1 developed AKI and anaemia (cluster 1), 1 developed AKI and 1 developed hypotension respectively (both were cluster 1, the latter was uncomplicated at presentation) (Fig. 2).

There were no deaths but two patients who were not recruited to the present study died during the study period; one identified posthumously as *P. knowlesi* by microscopy and another having been initially missed at presentation to Kapit Hospital but who was subsequently diagnosed at a tertiary hospital^29^. The latter case was confirmed as *P. knowlesi* by PCR and subsequently identified as cluster 1. Taking these two fatal cases into account, the overall fatality rate was 0.35% (2/572) (95% CI 0.04–1.3%).

## Discussion

In the present study, the clinical and laboratory features of patients infected with one of two divergent subpopulations of *P. knowlesi* in Kapit arising from different zoonotic hosts, one associated with long-tailed macaques (cluster 1) and the other with pig-tailed macaques (cluster 2)^9^, were prospectively characterised. Infections with each subpopulation resulted in a wide spectrum of disease but the clinical presentation was predominantly with uncomplicated malaria. Furthermore, the incidence of severe disease, the proportions of patients with individual criteria for severity, and the distribution of parasitaemia did not differ significantly between subpopulations. Haematological and biochemical findings revealed minor, clinically insignificant differences between subpopulations with cluster 1 infections having lower platelet counts and sodium serum concentrations, together with higher serum total bilirubin and urea concentrations, compared with cluster 2.

An ordinal logistic regression model was used to explore markers of end organ disease and found that, after adjusting for age, sex and ln (parasitaemia), cluster 1 had a greater association with renal dysfunction. A previous study in patients at Sibu and Sarikei in Sarawak has shown increased end organ disease with selected alleles in *P. knowlesi* subpopulations, including renal dysfunction^26^. In that study, the driver of a severe phenotype was an association with the *P. knowlesi* normocyte binding protein (PkNBP) xa gene, which along with the Duffy binding protein (DBP) gene, has been shown to be highly differentiated between the subpopulations^30^. PkNBP belongs to a family of invasion ligands proteins known as reticulocyte binding-like proteins that are associated with recognition of erythrocyte receptors upon which DBP proteins are released to begin invasion^31,32^. The dimorphism observed in these two genes do suggest plausible differences in efficiency of host cell selection and invasion between the two subpopulations. Disease severity in malaria is a complex interplay between the host’s immune system, parasite virulence and also entomological inoculation rate which contribute to the wide clinical spectrum commonly seen in malaria^33,34^. Therefore, clinical disease severity and virulence extend beyond heterogeneity in these genes which may explain the lack of distinct clinical phenotypes observed.

Consistent with previous prospective studies in Sarawak and Sabah^18,20^, severe disease was present in 10.5% of all knowlesi malaria cases regardless of subpopulation. Also consistent with previous reports^5,20,35^, AKI was the commonest severity criteria observed, while two or more severity criteria were present in half of our patients with severe disease. Anaemia following treatment occurred in eight of the patients. Post-treatment anaemia in uncomplicated malaria is well recognised and multifactorial, but has been attributed mainly to haemolysis of both parasitized and non-parasitized erythrocytes^36^. Delayed haemolysis has also been reported with artemisinin derivatives which could be a possible contributing factor in the present series^37,38^. This is thought to result from shorter lifespan in pitted erythrocytes following treatment and this has been found to correlate with hyperparasitaemia in falciparum malaria^37^.

The incidence of patients meeting severe respiratory criteria in the current study (n = 14/44, 32%) was higher than seen in a recent study from Sabah (n = 2/28, 7%)^20^. This may reflect between-study differences in diagnostic criteria. Overall there were no differences in severe phenotype between subpopulations but, since there were only eight severely ill patients with cluster 2 infection, more cases will need to be studied before definitive conclusions can be reached. The clinical characteristics of the seven patients infected with both subpopulations were not compared to the single cluster infections due to their small number. However, it is of interest that all were males with one severe case having 4 severe criteria including hyperparasitaemia (167,434/µL).

None of the patients recruited into the study died but, if two fatal knowlesi malaria cases that were known to have occurred during the study period were included, this would represent a mortality rate of 0.35%. This is lower, but not statistically different to, the rate of 1.8% observed during our first prospective study conducted 10 years ago at Kapit Hospital (*p* = 0.13)^18^. Despite an increased number of health staff, improved awareness of knowlesi malaria as a potential diagnosis, and prompt use of artemisinin-based combination therapies at Kapit Hospital compared with a decade ago, the two deaths that occurred were associated with delays in diagnosis^29^. Although late presentation associated with limited local transportation and health infrastructure may have been unavoidable, it was also frequently observed in the early descriptions of fatal cases^39^. As thrombocytopenia is almost universally detected at presentation in knowlesi malaria cases, it remains one of the most useful prompts for including *P. knowlesi* infection in the differential diagnosis of illness in endemic areas and in travellers returning from malaria-endemic countries^18^.

The age and sex distributions of the patients in the present study were similar to those previously described in the Kapit Division^18^, and the proportions of patients with cluster 1 and 2 infections were consistent with that of our previous study at approximately 70% and 30%, respectively^13^. Patients who had undertaken recent peri-domestic or forest fringe activities were more likely to have cluster 1 infections while those with cluster 2 infections reported recent jungle or interior forest exposure such as hunting and logging. The higher proportion of cluster 1 infections and the different risk factors of acquiring the two subpopulations could be due to the differences between the habitats preferred by the macaque hosts. Long-tailed macaques are adaptable to environmental changes and have a wide range of habitat, including being found in secondary forests close to human habitation^14,15^. In contrast pig-tail macaques prefer to live in primary forests away from human habitation^16^. It is also possible that there are differences in the mosquito species responsible for transmission of each of the two subpopulations. Detailed studies on the bionomics of vectors and the distribution and relative abundance of the two different macaque hosts need to be undertaken for a comprehensive understanding of the transmission dynamics of the two *P. knowlesi* subpopulations in Malaysian Borneo.

In conclusion, infections with the two divergent subpopulations of *P. knowlesi* present in Kapit resulted in similar disease phenotypes, and so testing for cluster type at presentation is unlikely to alter clinical management. Although they result in predominantly uncomplicated malaria, both clusters can lead to potentially life threatening complications. The population risk profile differs between the two subpopulations, and cluster 1 infections may cause severe disease across a wider range of parasitaemia and are more likely associated with renal dysfunction. Despite increased awareness and improved healthcare compared to the first prospective study 10 years ago, the knowlesi malaria fatality rate has not changed. Clinicians working in Southeast Asia and those managing travellers returning from *P. knowlesi* endemic areas thus need to be aware of the wide spectrum of disease and risk of potentially life-threatening complications in knowlesi malaria.

## Methods

### Study site and subjects

A prospective observational study was conducted at Kapit Hospital, in the Kapit Division of Sarawak, Malaysian Borneo. Kapit Division covers an area of 38,934 km^2^ with a total population of 112,762 and mainly of Iban ethnicity (67.4%)^40^. All individuals who are blood slide microscopy-positive for malaria are required to be hospitalised in Malaysia and discharged when clinically well and have had two malaria-negative blood films on two consecutive days.

All non-pregnant patients aged ≥ 15 years admitted to Kapit Hospital between September 2016 and October 2018 with a positive blood film for either *P. knowlesi* or *P. malariae* and no travel history outside of Malaysia within the previous 28 days were eligible for enrolment. Patients with significant co-morbidities (established end organ disease or co-infection) or who had received antimalarial treatment within the previous 14 days were excluded. Patients diagnosed at a peripheral health care facility were included if they arrived at Kapit Hospital within one hour of starting antimalarial treatment. Only patients with a PCR confirmed *P. knowlesi* monoinfection were subsequently included in the study. All patients provided informed written consent to the study procedures which were approved by the Medical Research Ethics Committee of Universiti Malaysia Sarawak and the Medical Research and Ethics Committee of the Ministry of Health Malaysia (NMRR-16-943-31224 [IIR])**.** All procedures on patients were performed in accordance with relevant guidelines and regulations.

### Epidemiology data and clinical procedures

Patient demographics, recent travel history including activities within 14 days prior to hospital admission, clinical details and baseline laboratory data were recorded on a standardised case report form by study clinicians. Recent peri-domestic activities were defined as any outdoor activities that were conducted around the perimeter of residential households and towards the town centre, while recent jungle activities refer to any outdoor activities inside deep jungle including logging, activities around the logging camps, jungle trekking and hunting. Ten mL of venous blood was collected from each patient for biochemical and haematologic tests. Severe knowlesi malaria disease was defined according to the WHO research criteria 2014^27^ at the time of, or during course of admission. The criteria were: hyperparasitaemia (> 100,000 parasites/μL); severe anaemia (haemoglobin concentration < 7 g/dL); hypotension (systolic blood pressure < 80 mmHg); AKI (serum creatinine > 265 μmol/L or blood urea > 20 mmol/L); jaundice (serum bilirubin > 50 μmol/L) with parasite count > 20,000/μL; hypoglycaemia (serum glucose < 2.2 mmol/L); acidosis (base deficit > 8 mEq/L or plasma bicarbonate < 15 mmol/L or venous plasma lactate ≥ 5 mmol/L); acute pulmonary oedema/acute respiratory distress syndrome (ARDS) (respiratory rate > 30 breaths/minute plus oxygen saturation < 92% on air and/or pulmonary infiltrates on chest radiograph); cerebral malaria (unarousable coma). In assessing the presence of acidosis, serum bicarbonate or base excess was used when plasma lactate was unavailable, or vice versa, as per WHO 2014 research criteria.

Treatment followed the Malaysian Ministry of Health (MMoH) guidelines, including the diagnosis of severe malaria in patients with > 20,000 parasites/µL^41^. All patients received artemether–lumefantrine and/or intravenous artesunate depending on disease severity. Clinical management, including the investigation and treatment of bacterial infection, was otherwise at the discretion of the attending clinician. Similarly, each patient was assessed at least daily by the treating team and baseline investigations only repeated if clinically indicated.

### Laboratory procedures

Blood spots were prepared on filter paper from the blood sample taken from each patient and subsequently processed at the Malaria Research Centre, Universiti Malaysia Sarawak. DNA was extracted from the blood spots using InstaGene™^42^ and nested PCR assays for identification of malaria species with primers for *P. falciparum, P. malariae, P. vivax, P. ovale, P. knowlesi, P. cynomolgi* and *P. inui* were utilised as described previously^1,43,44^. Each *P. knowlesi* subpopulation was identified using cluster-specific PCR assays^13^. Parasite counts were determined using the average readings of two independent experienced laboratory technicians derived from the number of parasites per 500 white cells, adjusted by the total white cell count for each patient.

Haematological profiles on-site at the hospital laboratory were determined using semi-automated methods (Mek-6410 K, Nihon Kohden Corporation, Japan). Citrated blood samples were analysed for coagulation studies with Sysmex® Ca 500v (Kobe, Japan). Biochemistry tests were conducted using Beckman-Coulter™ AU480 and a point-of-care meter was used for determination of lactate levels (StatStrip Xpress® Lactate Hospital Meter, Nova Biomedical, USA).

### Statistical analyses

All analyses were carried out in R Studio version 1.2.1335^45^ using arsenal, finalfit, pROC, cutpointr, ggplot2 and ggupset packages. Pairwise deletion was used in analysing the data with number of observations for each variable listed in Supplementary Table S6 online. Normally and non-normally distributed continuous data were compared using independent *t*-test and Mann–Whitney test respectively. Parasite densities were natural logarithm (ln) transformed before analysis. Proportions were compared using Fisher’s exact test with post hoc pairwise comparison using Bonferroni correction. Ordinal logistic regression was used to detect differences between subpopulations with normal, abnormal and defining criteria for severe disease severity, and adjustment for confounders. The categorisation of variables used in ordinal logistic regression was normal (1), abnormal but not severe (2) and severe (3) as summarised in Supplementary Table S7 online. Logistic and continuous linear regression were used to determine associations with outcome after adjusting for confounders. In continuous linear regression, non-normal data was transformed with natural logarithm (ln). Receiver-operating characteristic (ROC) and area under the ROC curve (AUC) were used to determine reliability of predictors and Youden’s index (equal weight was given to sensitivity and specificity) was used to determine optimal cut-off for variables. The optimal cut-off for these variables was analysed in multivariable logistic regression controlled for age, sex and ln (parasitaemia). All analyses considered a *p* value of < 0.05 (two-tailed) as significant.

## Supplementary Information

Supplementary Tables.
