# Supplementary material for: A comparison of the clinical, laboratory and epidemiological features of two divergent subpopulations of Plasmodium knowlesi
Source: Sci Rep. 2021 Oct 11;11:20117. doi: 10.1038/s41598-021-99644-8 (PMC8505493; doi:10.1038/s41598-021-99644-8)
Supplement: Supplementary file 1 — Supplementary Tables. [file 41598_2021_99644_MOESM1_ESM.docx]

A comparison of the clinical, laboratory and epidemiological features of two divergent subpopulations of *Plasmodium knowlesi*

Ting Huey Hu^1^, Nawal Rosli^1^, Dayang A. Mohamad^1^, Khamisah A. Kadir^1^, Zhen Hao Ching^2^, Yaw Hung Chai^2^, Nur Naqibah Ideris^2^, Linda S.C. Ting^2^, Adeline A. Dihom^2^, Sing Ling Kong^2^, Edmund K.Y. Wong^2^, Jenny E.H. Sia^2^, Tiana Ti^2^, Irene P.F. Chai^2^, Wei Yieng Tang^2^, King Ching Hii^2^, Paul C.S. Divis^1^, Timothy M.E. Davis^1,3^, Cyrus Daneshvar^1,4^ and Balbir Singh^1^*

^1^Malaria Research Centre, Universiti Sarawak Malaysia, Kota Samarahan, Malaysia

^2^Kapit Hospital, Kapit, Sarawak, Malaysia

^3^University of Western Australia, Medical School, Fremantle, Western Australia, Australia

^4^Department of Respiratory Medicine, University Hospitals Plymouth NHS Trust, Plymouth, UK

*bsingh@unimas.my

| **Dependent: Types of activities** | | **Jungle activity (baseline)** | **Peri-domestic activity** | **OR (95% CI) (univariable)** | **P-value** | **OR (95% CI) (multivariable)** | **P-value** |
| --- | --- | --- | --- | --- | --- | --- | --- |
| Sex | Male | 142 (84.5) | 105 (44.3) | 1 |  | 1 |  |
|  | Female | 26 (15.5) | 132 (55.7) | 6.87 (4.26-11.4) | **<0.001** | 5.09 (3.09-8.62) | **<0.001** |
| Age, years | Mean (SD) | 36.0 (13.1) | 49.4 (18.3) | 1.05 (1.04-1.07) | **<0.001** | 1.04 (1.03-1.06) | **<0.001** |

**Supplementary Table S1.** Association of the types of activity with sex and age in *P. knowlesi* patients (N=405). Data are presented as no. (%). P<0.05 in bold.

| **Linear regression** | **cluster 1 (cluster 2 as baseline)** | | | |
| --- | --- | --- | --- | --- |
| **Outcome** | **Coefficient (95%CI)**  **(univariable)** | **P value** | **Coefficient (95%CI) (multivariable)** | **P value** |
| Ln prothrombin time (s) | 0.04 (-0.01 to 0.08) | 0.105 | 0.05 (0.00 to 0.09) | **0.042** |
| Ln platelet count (platelet/µL) | -0.24 (-0.38 to -0.09) | **0.001** | -0.25 (-0.38 to -0.12) | **<0.001** |
| Ln serum urea level (mmol/L) | 0.20 (0.08 to 0.32) | **0.001** | 0.19 (0.10 to 0.29) | **<0.001** |
| Ln serum total bilirubin level (µmol/L) | 0.13 (0.00 to 0.27) | **0.048** | 0.17 (0.05 to 0.28)§ | **0.005** |
| Serum sodium level (mmol/L) | -1.06 (-1.95 to -0.16) | **0.021** | -1.08 (-1.91 to -0.25) | **0.011** |
| Ln parasitaemia (parasites/µL) | -0.02 (-0.49 to 0.44) | 0.922 | -0.11 (-0.53 to 0.31) | 0.596 |

**Supplementary Table S2**. Summary of association between *P. knowlesi* clusters and univariate associates in linear regression (cluster 1 =298, cluster 2= 115, N=413). Adjusted for age, sex and ln (parasitaemia) (except when ln (parasitaemia) as continuous outcome, then adjusted for severe disease. Serum sodium level remained significant (P=0.037) after adjusted for referral. P<0.05 in bold.

| **Variables** | | **Cluster 1** | **Cluster 2**  **(baseline)** | **OR (95% CI) (univariable)** | **P-value** | **OR (95% CI) (multivariable)** | **P-value** | **ROC analysis** | | | | | |
| --- | --- | --- | --- | --- | --- | --- | --- | --- | --- | --- | --- | --- | --- |
|  | |  |  |  |  |  |  | **Sens** | **Specs** | **PPV** | **NPV** | **AUC** | **95% CI** |
| Platelet count | >50 x10^3^/µL | 160 (61.8) | 81 (83.5) | 1 | **-** | 1 | **-** |  |  |  |  |  |  |
|  | ≤50 x10^3^/µL | 99 (38.2) | 16 (16.5) | 3.13 (1.77-5.83) | <0.001 | 3.93 (2.14-7.63) | **<0.001** | 38 | 84 | 86.6 | 32.8 | 0.62 | 0.55,0.68 |
| Serum sodium | >131 mmol/L | 156 (60.2) | 69 (71.1) | 1 |  | 1 |  |  |  |  |  |  |  |
|  | ≤131 mmol/L | 103 (39.8) | 28 (28.9) | 1.63 (0.99-2.73) | 0.059 | 1.74 (1.04-2.97) | **0.040** | 40 | 71 | 78.6 | 30.7 | 0.44 | 0.37,0.55 |
| Serum total bilirubin | <25 µmol/L | 149 (57.5) | 70 (72.2) | 1 | - | 1 | **-** |  |  |  |  |  |  |
|  | ≥25 µmol/L | 110 (42.5) | 27 (27.8) | 1.91 (1.16-3.22) | 0.012 | 2.28 (1.33-4.01) | **0.003** | 42 | 75 | 82.0 | 32.7 | 0.57 | 0.50,0.63 |
| Serum urea | <5.4 mmol/L | 146 (54.3) | 71 (73.2) | 1 | **-** | 1 | **-** |  |  |  |  |  |  |
|  | ≥5.4 mmol/L | 123 (45.7) | 26 (26.8) | 2.30 (1.40-3.88) | 0.001 | 3.09 (1.73-5.70) | **<0.001** | 46 | 73 | 82.6 | 32.7 | 0.62 | 0.55，0.68 |

**Supplementary Table S3**. Receiver-operating characteristic (ROC) and area under ROC curve (AUC) analyses of predictors for clusters

Adjusted for age, sex and ln (parasitaemia). Sens= sensitivity, Specs=specificity, PPV=positive predictive value, NPV=negative predictive value.

|  | **Cluster 1 (Cluster 2 as baseline)** | | | |
| --- | --- | --- | --- | --- |
| **Complications** | **OR (95%CI) (univariable)** | **P-value** | **OR (95%) (multivariable)** | **P-value** |
| ARDS | 1.76 (0.44-11.66) | 0.474 | 1.53 (0.35-10.62) | 0.608 |
| Hyperparasitaemia | 1.16 (0.40-4.23) | 0.796 | 1.00 (0.34-3.69) | 0.994 |
| Hypotension | 1.16 (0.15-23.57) | 0.899 | 0.87 (0.10-18.67) | 0.906 |
| AKI | 3.21 (0.89-20.49) | 0.124 | 3.90 (0.90-27.99) | 0.105 |
| Jaundice | 1.27 (0.44-4.57) | 0.686 | 1.82 (0.34-11.76) | 0.496 |
| Acidosis | 1.76 (0.44-11.66) | 0.474 | 2.30 (0.48-17.39) | 0.343 |

**Supplementary Table S4**. Association between severe criteria and *P. knowlesi* clusters (cluster 1, N=298; cluster 2, N = 115) adjusted for age, sex and ln (parasitaemia).

| **Dependent: Severe Disease** | | **False** | **True** | **OR (95% CI) (univariable)** | **P-value** | **OR (95% CI) (multivariable)** | **P-value** |
| --- | --- | --- | --- | --- | --- | --- | --- |
| Clusters, no. (%) | cluster 2 | 106 (28.6) | 9 (20.9) | 1 | - | 1 | **-** |
|  | cluster 1 | 264 (71.4) | 34 (79.1) | 1.52 (0.73-3.46) | 0.288 | 1.48 (0.58-4.11) | 0.427 |
| Ln ( parasitaemia) | Mean (SD) | 6.9 (1.9) | 10.2 (2.2) | 2.90 (2.30-3.78) | **<0.001** | 2.94 (2.31-3.90) | **<0.001** |
| Age, years | Mean (SD) | 42.8 (17.3) | 54.7 (16.6) | 1.04 (1.02-1.06) | **<0.001** | .03 (1.01-1.06) | **0.007** |
| Sex, no. (%) | Female | 135 (36.5) | 27 (62.8) | 1 | - | 1 | - |
|  | Male | 235 (63.5) | 16 (37.2) | 0.34 (0.17-0.65) | **<0.001** | 0.57 (0.24-1.34) | 0.198 |

**Supplementary Table S5.** Association between severe disease and *P. knowlesi* clusters (cluster 1, N=298; cluster 2, N = 115). P<0.05 in bold.

| **Variables** | **Cluster 1 (N=298)** | **Cluster 2 (N=115)** |
| --- | --- | --- |
|  | **No of observations** | **No of observations** |
| Age | 298 | 115 |
| Duration of illness (Days) | 298 | 115 |
| Pulse Rate (beats/min) | 298 | 115 |
| Respiratory Rate (breaths/min) | 298 | 115 |
| Mean arterial pressure (mm/Hg) | 298 | 115 |
| Oxygen saturations (%) | 298 | 115 |
| Haemoglobin (g/dL) | 290 | 106 |
| White blood cell count (x10^3^/µL) | 289 | 106 |
| Platelet count (x10^3^/µL) | 289 | 106 |
| Prothrombin time (s) | 287 | 113 |
| Serum creatinine (µmol/L) | 290 | 109 |
| Serum urea (mmol/L) | 285 | 112 |
| Serum total bilirubin (µmol/L) | 294 | 110 |
| Serum alanine aminotransferase (IU/L) | 292 | 112 |
| Serum albumin (g/dL) | 290 | 110 |
| Serum sodium (mmol/L) | 290 | 113 |
| Parasite count (parasites/µL) | 298 | 115 |
| Serum glucose (mmol/L) | 295 | 115 |
| Plasma lactate (mmol/L) | 240 | 93 |

**Supplementary Table S6**. Number of observations for each variable used in univariate analysis.

| **Variables** | **Normal (1)** | **Abnormal but not severe (2)** | **Severe (3)** |
| --- | --- | --- | --- |
| Anaemia, haemoglobin, g/dL | ≥10 | 7-9 | <7 |
| Abnormal liver function, serum alanine aminotransferase (ALT), U/L | ≤ 60 | 61-500 | >500 |
| Renal dysfunction, blood urea mmol/L or serum creatinine µmol/L | ≤ 8.3 or ≤ 133 | 8.4-20 or 134-265 | >20 or >265 |
| Hyperbilirubinaemia, serum total bilirubin, µmol/L | ≤20 regardless of parasite count | >20 plus parasite count ≤20,000/µL or 21-50 plus parasite count >20,000/µL | >50 plus parasite count >20,000/µL |
| Acidaemia, lactate acid, mmol./L | <2.5 | 2.5-4.9 | ≥5 |
| Respiratory dysfunction, breath per minutes (bpm) plus oxygen saturation (%) | ≤25 bpm plus ≥92% on air and no pulmonary oedema | 26-30 bpm plus ≥92% on air and no pulmonary oedema or <30 bpm plus <92% on air and no pulmonary edema or >30 bpm plus ≥92% on air and no pulmonary edema | >30bpm plus <92% on air and/or pulmonary oedema |
| Haemodynamic stability, systolic BP (mmHg) | ≥ 100 | 80-99 | <80 |

**Supplementary Table S7**. Categorisation of variables used in ordinal logistic regression to differentiate between subpopulations in disease severity
